# Supplementary material for: Classifying high-dimensional phenotypes with ensemble learning
Source: bioRxiv. 2023 May 29:2023.05.29.542750. Preprint. [Version 1] doi: 10.1101/2023.05.29.542750 (PMC10312448; doi:10.1101/2023.05.29.542750)
Supplement: Supplement 1 [file media-1.pdf]

Table S1. Complete summary of phenotypic datasets, including the family, landmarked anatomy, total sample size (*N*), class, number of class levels, number of landmarks (*p*), the dimensionality (*k*), and the original data repository.

Family	Anatomy	*N*	Class	Levels	*p*	*k*	Data Repository
Asterinidae	Body	885	Sex	2	10	2	http://dx.doi.org/10.5061/dryad.4h31p
Drosophilidae (a)	Wing	2926	Sex	2	48	2	http://dx.doi.org/10.5061/dryad.r43k1.2
Emydidae	Shell	2161	Habitat	2	53	3	https://doi.org/10.5061/dryad.8r0m76h
Gasterosteidae (1)	Skull	190	Habitat	2	70	3	https://doi.org/10.5061/dryad.xd2547dkw
Gasterosteidae (2)	Body	521	Sex	2	15	2	https://doi.org/10.5061/dryad.bzkh189cx
Hominidae	Sacrum	101	Sex	2	100	3	https://doi.org/10.1371/journal.pone.0264770
Hynobiidae/Cryptobranchidae	Palate	62	Habitat	2	24	2	https://doi.org/10.5061/dryad.c59zw3r8x
Muridae (a)	Cranium	1251	Sex	2	844	3	https://doi.org/10.1038/s41597-022-01338-x
Poeciliidae	Body	1449	Sex	2	13	2	https://doi.org/10.5061/dryad.2sh53
Serranidae/Sparidae	Body	259	Site	2	13	2	https://doi.org/10.5061/dryad.1k571
Cichlidae	Jaw	1136	Tribe	14	42	3	https://doi.org/10.5061/dryad.9w0vt4bbf
Colubridae+	Vertebrae	1260	Species	15	12	2	https://doi.org/10.5061/dryad.jq285
Crocodylidae/Alligatoridae	Cranium	183	Species	8	78	3	http://dx.doi.org/10.5061/dryad.14fn1
Drosophilidae (b)	Wing	2926	Elevation	9	48	2	http://dx.doi.org/10.5061/dryad.r43k1.2
Formicidae	Face	1494	Species	6	11	2	http://dx.doi.org/10.5061/dryad.f65bn
Muridae (b)	Cranium	1251	Genotype	26	844	3	https://doi.org/10.1038/s41597-022-01338-x
Ocypodidae	Carapace	1867	Species	16	21	2	http://dx.doi.org/10.5061/dryad.vv197
Percidae	Body	423	Species	15	10	2	https://doi.org/10.5061/dryad.n28rf
Vespidae	Wing	206	Species	8	19	2	http://dx.doi.org/10.5061/dryad.4588r
Viviparidae	Shell	1224	Population	22	127	2	https://doi.org/10.5061/dryad.vm523

Table S2. Summary of performance metrics for each method among all binary class datasets.

Method	F1	Accuracy	Sensitivity	Specificity	Precision	Kappa
AdaBag	0.86	0.84	0.88	0.81	0.85	0.7
AdaBoost.M1	0.9	0.88	0.9	0.87	0.9	0.78
bagEarthGCV	NA	0.13	NA	NA	0.14	NA
C5.0	0.88	0.87	0.89	0.85	0.88	0.75
cforest	0.88	0.86	0.9	0.81	0.87	0.73
earth	0.85	0.85	0.85	0.84	0.85	0.69
evtree	0.84	0.81	0.87	0.75	0.83	0.63
fda	0.84	0.84	0.83	0.85	0.86	0.69
gaussprLinear	0.85	0.86	0.83	0.89	0.88	0.68
gaussprPoly	0.79	0.77	0.74	0.8	0.84	0.55
gaussprRadial	0.86	0.83	0.83	0.83	0.9	0.68
glmnet	0.87	0.83	0.89	0.77	0.86	0.67
hda	0.9	0.9	0.89	0.91	0.92	0.8
hdda	0.87	0.87	0.87	0.86	0.87	0.72
kernelpls	0.89	0.87	0.9	0.85	0.88	0.75
kknn	0.86	0.85	0.89	0.82	0.86	0.71
lda	0.89	0.88	0.88	0.87	0.9	0.76
loclda	0.9	0.9	0.89	0.9	0.91	0.79
LogitBoost	0.86	0.84	0.86	0.82	0.86	0.68
mda	0.89	0.89	0.88	0.9	0.9	0.78
nb	0.88	0.87	0.87	0.86	0.88	0.74
nnet	0.9	0.89	0.91	0.88	0.9	0.79
pda	0.89	0.88	0.89	0.86	0.89	0.76
pls	0.89	0.88	0.89	0.87	0.9	0.77
qda	0.89	0.88	0.88	0.87	0.9	0.76
ranger	0.87	0.84	0.88	0.8	0.86	0.7
rda	0.91	0.91	0.91	0.91	0.91	0.8
sparseLDA	0.89	0.88	0.9	0.86	0.89	0.76
stepLDA	0.89	0.87	0.88	0.87	0.89	0.76
stepQDA	0.82	0.78	0.83	0.73	0.82	0.57
svmLinear	0.89	0.88	0.88	0.87	0.89	0.76
svmPoly	0.89	0.9	0.87	0.92	0.92	0.8
svmRadial	0.89	0.89	0.89	0.88	0.89	0.78
top_10_glm_ens	0.9	0.9	0.9	0.91	0.92	0.81
top_10_rf_ens	0.9	0.89	0.89	0.89	0.9	0.79
top_3_glm_ens	0.85	0.81	0.76	0.85	0.94	0.62
top_3_rf_ens	0.91	0.91	0.92	0.9	0.91	0.82
top_5_glm_ens	0.85	0.81	0.82	0.81	0.87	0.63
top_5_rf_ens	0.91	0.9	0.9	0.91	0.91	0.81
treebag	0.86	0.84	0.88	0.8	0.85	0.69

xgbDART	0.86	0.85	0.86	0.84	0.88	0.7

Table S3. Summary of performance metrics for each method among all multi-class datasets.

**Method**	**F1**	**Accuracy**	**Sensitivity**	**Specificity**	**Precision**	**Kappa**
AdaBag	0.68	0.8	0.64	0.97	0.72	0.67
AdaBoost.M1	0.73	0.84	0.69	0.98	0.74	0.72
bagEarthGCV	0.76	0.85	0.72	0.98	0.77	0.73
C5.0	0.68	0.81	0.65	0.97	0.7	0.67
cforest	0.72	0.81	0.64	0.97	0.73	0.68
earth	0.71	0.82	0.67	0.97	0.73	0.69
evtree	0.6	0.74	0.52	0.96	0.58	0.55
fda	0.72	0.83	0.69	0.97	0.73	0.7
glmnet	0.76	0.86	0.74	0.98	0.77	0.75
hda	0.71	0.83	0.69	0.97	0.73	0.7
hdda	0.73	0.84	0.71	0.98	0.73	0.71
kernelpls	0.68	0.78	0.59	0.97	0.73	0.66
kknn	0.76	0.85	0.73	0.98	0.76	0.73
lda	0.76	0.86	0.74	0.98	0.76	0.75
loclda	0.73	0.84	0.71	0.98	0.72	0.71
LogitBoost	0.73	0.82	0.67	0.97	0.72	0.69
mda	0.79	0.88	0.78	0.98	0.79	0.78
nb	0.7	0.83	0.68	0.97	0.73	0.69
nnet	0.76	0.85	0.73	0.98	0.76	0.73
pda	0.76	0.86	0.74	0.98	0.76	0.75
pls	0.68	0.78	0.59	0.97	0.74	0.66
ranger	0.75	0.85	0.72	0.98	0.79	0.74
rda	0.71	0.84	0.7	0.98	0.71	0.69
sparseLDA	0.76	0.86	0.74	0.98	0.76	0.75
stepLDA	0.75	0.86	0.74	0.98	0.75	0.74
stepQDA	0.58	0.74	0.52	0.96	0.58	0.51
svmLinear	0.51	0.68	0.41	0.94	0.45	0.34
svmPoly	0.52	0.68	0.41	0.95	0.45	0.35
svmRadial	0.54	0.68	0.42	0.95	0.46	0.37
top_10_glm_ens	0.82	0.88	0.79	0.98	0.8	0.79
top_10_rf_ens	0.8	0.88	0.78	0.98	0.8	0.78
top_3_glm_ens	0.81	0.88	0.78	0.98	0.81	0.78
top_3_rf_ens	0.81	0.88	0.78	0.98	0.79	0.78
top_5_glm_ens	0.81	0.88	0.79	0.98	0.83	0.79
top_5_rf_ens	0.8	0.88	0.78	0.98	0.79	0.79
treebag	0.68	0.8	0.62	0.97	0.68	0.65
xgbDART	0.65	0.74	0.52	0.96	0.62	0.56

Table S4. Average relative rank within datasets.

**Method**	**Binary**	**Multi**
AdaBag	-0.04	0.42
AdaBoost.M1	-0.45	0.03
bagEarthGCV	1	-0.19
C5.0	-0.13	0.38
cforest	0.08	0.28
earth	-0.07	0.28
evtree	0.08	0.76
fda	0.12	0.21
gaussprLinear	0	NA
gaussprPoly	0.11	NA
gaussprRadial	-0.13	NA
glmnet	-0.13	-0.28
hda	-0.39	0.17
hdda	-0.23	-0.21
kernelpls	-0.23	0.3
kknn	-0.04	-0.18
lda	-0.23	-0.25
loclda	-0.5	-0.58
LogitBoost	0.29	-0.03
mda	-0.29	-0.44
nb	0.08	0.17
nnet	-0.54	-0.24
pda	-0.26	-0.29
pls	-0.25	0.29
qda	-0.26	-0.5
rf	-0.05	-0.2
rda	-0.61	-0.44
sparseLDA	-0.29	-0.32
stepLDA	-0.21	-0.19
stepQDA	0.42	0.64
svmLinear	-0.12	0.91
svmPoly	-0.18	0.87
svmRadial	-0.55	0.87
top_10_glm_ens	-0.61	-0.8
top_10_rf_ens	-0.5	-0.6
top_3_glm_ens	-0.19	-0.66
top_3_rf_ens	-0.72	-0.68
top_5_glm_ens	-0.38	-0.73
top_5_rf_ens	-0.7	-0.61
treebag	-0.01	0.37
xgbDART	-0.21	0.64

Table S5. Summary of phenotypic and dataset variable means, effect sizes, and covariate task effect sizes in each balanced accuracy regression, as well as the model standard error (SE), F statistic, and overall R^2.

**Variable**	**Binary**	**Multi**	**Variable Effect**	**Task Effect**	**SE**	**F**	$\mathbf{R^2}$
R^2	0.2	0.5	4.1%	-17.2%	9.1	7.7	0.41
Shape distance	0.06	0.08	1.4%	-7.4%	10.1	4.8	0.29
Covariance distance	0.1	0.2	-2.1%	NA	10.8	4.9	0.17
Variance ratio	1.2	1.5	0.3%	-6.2%	10.8	3.0	0.17
Class balance	0.9	0.9	-0.5%	NA	12.2	0	-0.05
Sample size	981	1197	0%	NA	12.2	0.1	-0.05

Table S6. Summary of average validation and test performance among all methods but within datasets.

**Dataset**	**Task**	**Vali F1**	**Test F1**	**Vali Accuracy**	**Test Accuracy**
Asterinidae	Binary	0.99	0.99	0.95	0.97
Drosophilidae (a)	Binary	0.85	0.90	0.87	0.91
Emydidae	Binary	0.94	0.97	0.82	0.85
Gasterosteidae (1)	Binary	1.00	0.99	0.97	0.99
Gasterosteidae (2)	Binary	0.90	0.86	0.91	0.87
Hominidae	Binary	0.53	0.78	0.54	0.79
Hynobiidae	Binary	0.96	0.98	0.91	0.97
Muridae (a)	Binary	0.84	0.85	0.82	0.82
Poeciliidae	Binary	0.89	0.90	0.85	0.86
Serranidae	Binary	0.49	0.55	0.55	0.60
Cichlidae	Multi	0.82	0.87	0.88	0.92
Colubridae+	Multi	0.84	0.87	0.89	0.92
Crocodylidae	Multi	0.86	0.89	0.89	0.91
Drosophilidae (b)	Multi	0.49	0.54	0.70	0.73
Formicidae	Multi	0.63	0.62	0.78	0.77
Muridae (b)	Multi	0.73	0.74	0.84	0.85
Ocypodidae	Multi	0.88	0.88	0.93	0.93
Percidae	Multi	0.62	0.54	0.76	0.72
Vespidae	Multi	0.82	0.88	0.89	0.92
Viviparidae	Multi	0.34	0.32	0.55	0.56

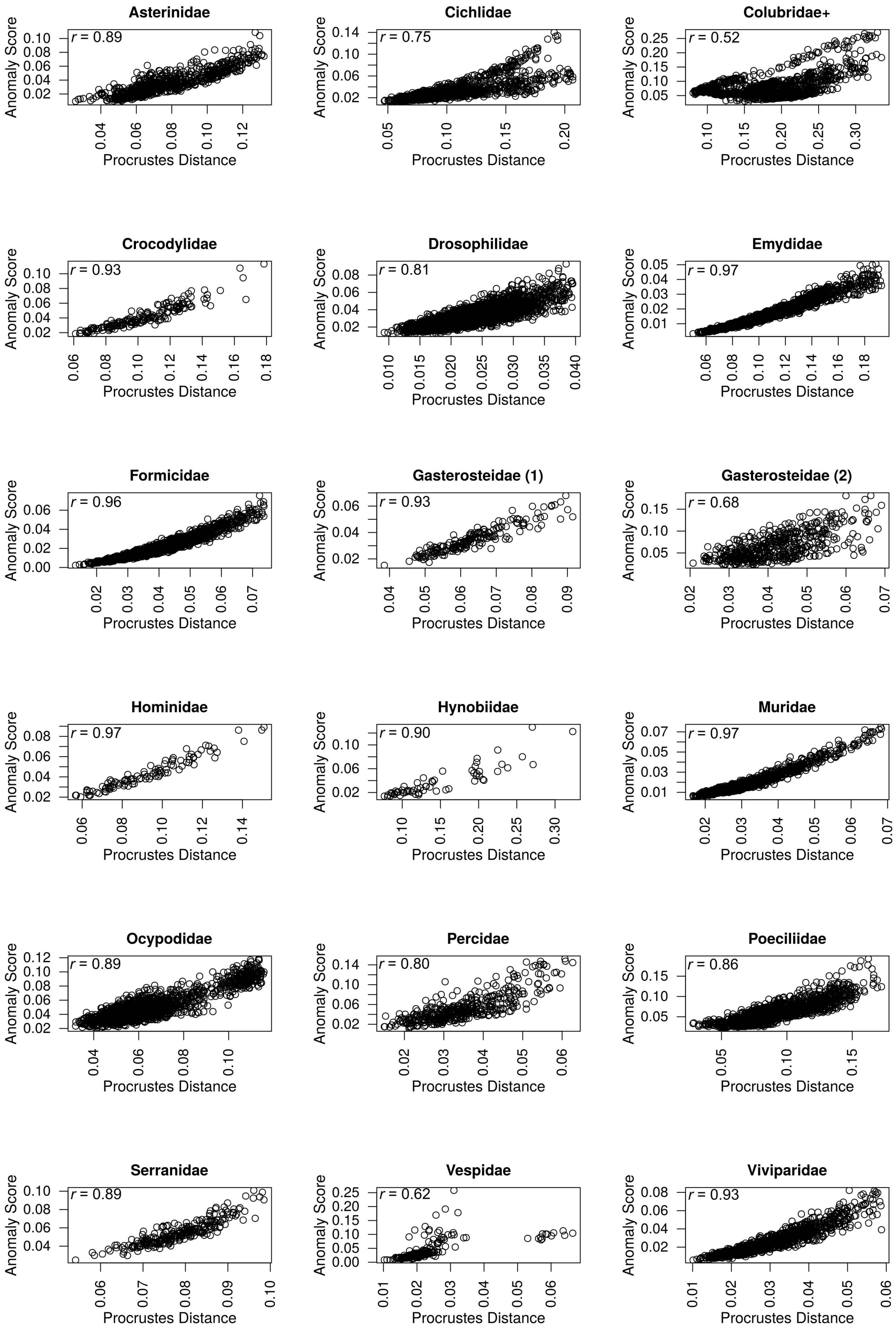

Figure S1. Bivariate plots for autoencoder anomaly scores and Procrustes distances to the mean. Correlation coefficients (*r*) are included. Plots are organized alphabetically by dataset.

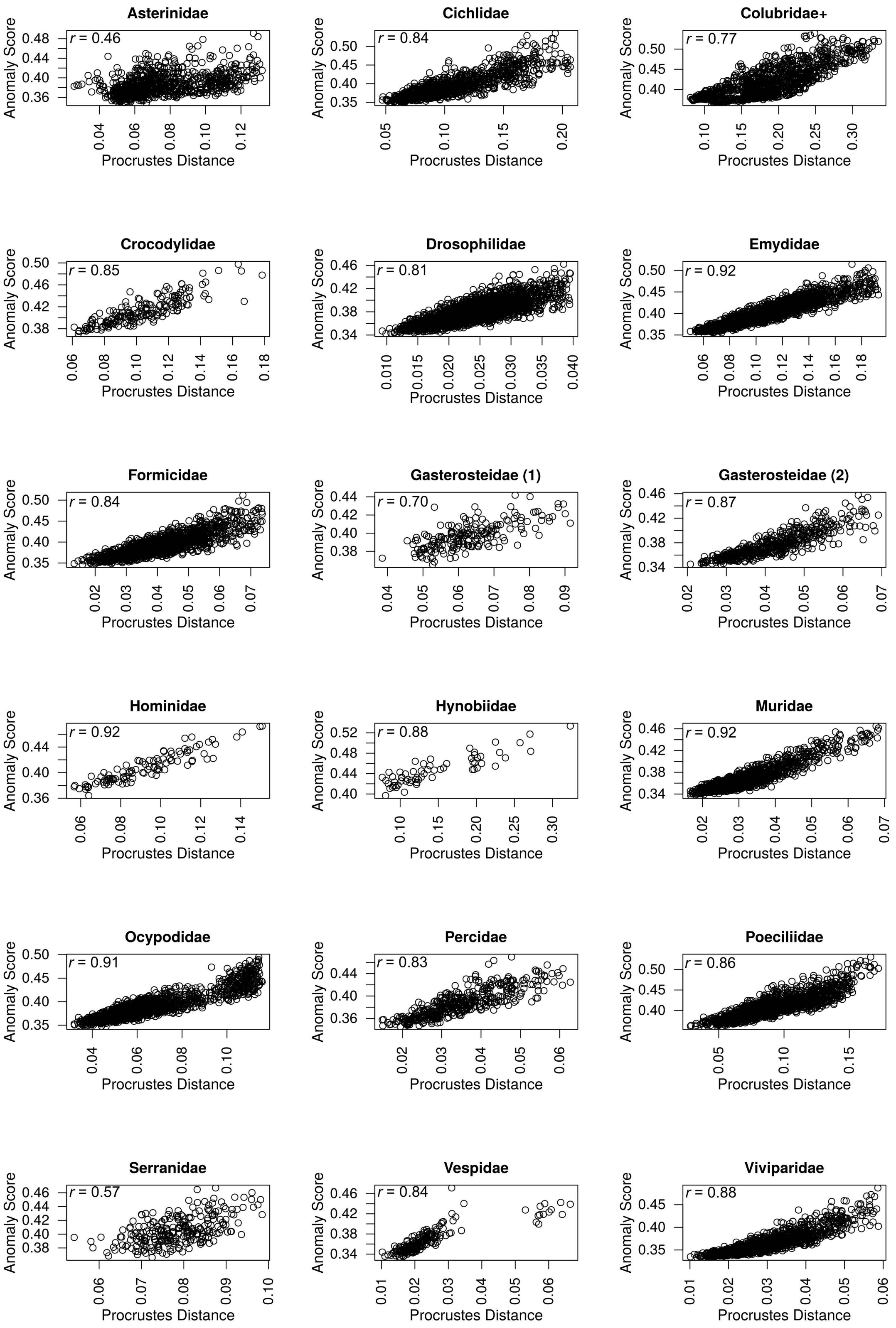

Figure S2. Bivariate plots for extended isolation forest anomaly scores and Procrustes distances to the mean. Correlation coefficients (*r*) are included. Plots are organized alphabetically by dataset.

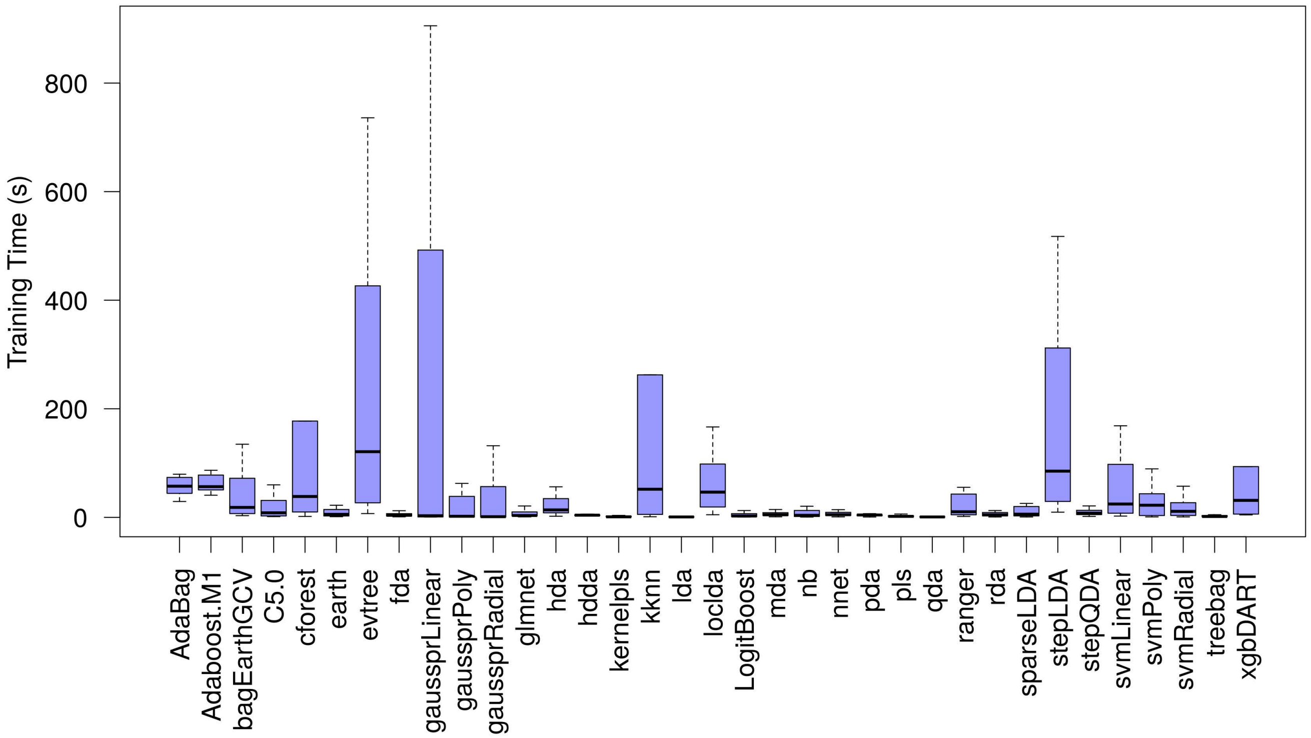

Figure S3. Distribution of training times (in seconds) for each method ordered alphabetically.

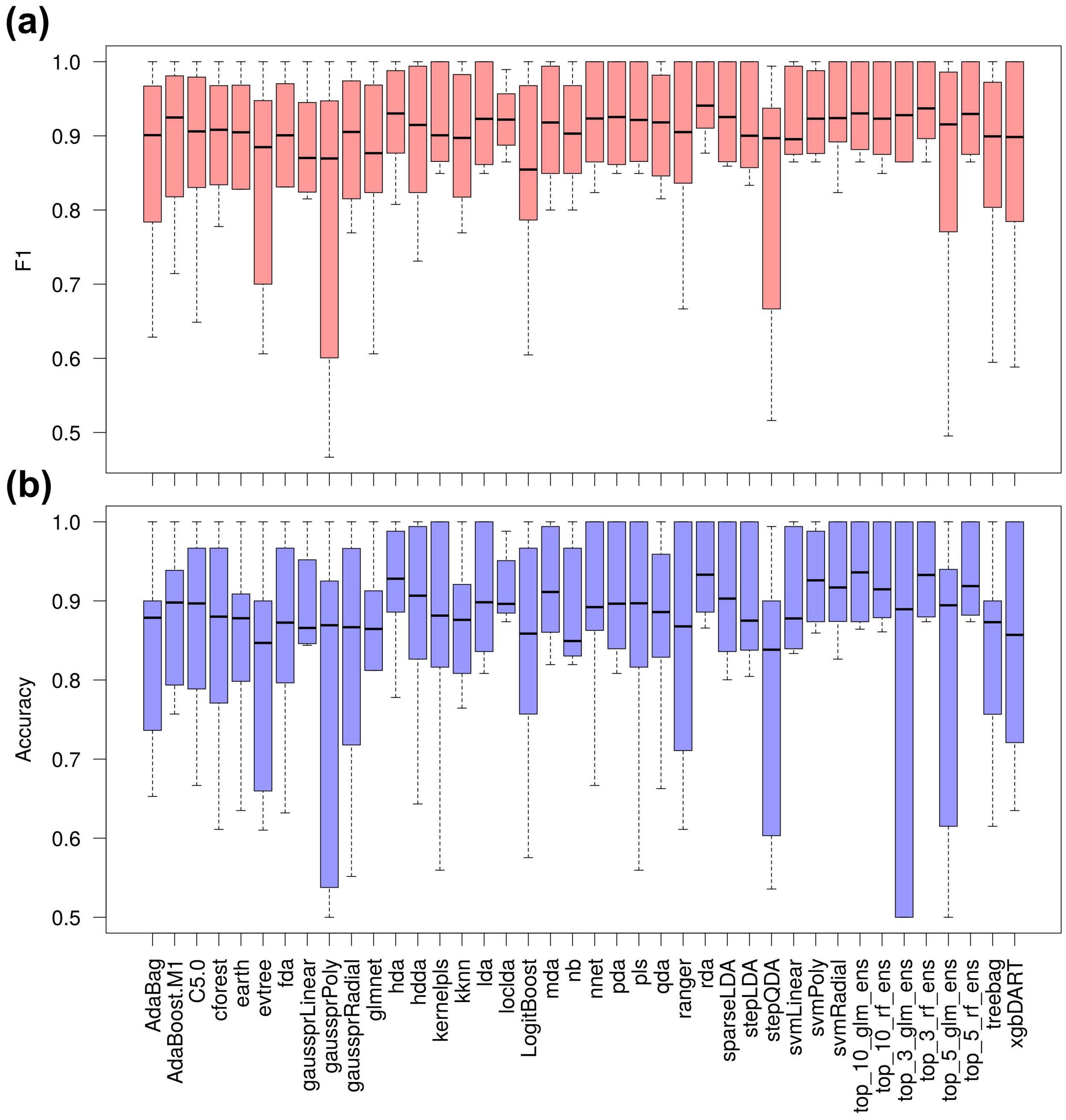

Figure S4. Distribution of (a) F1 and (b) balanced accuracy scores for every method among binary class datasets. Methods are ordered alphabetically.

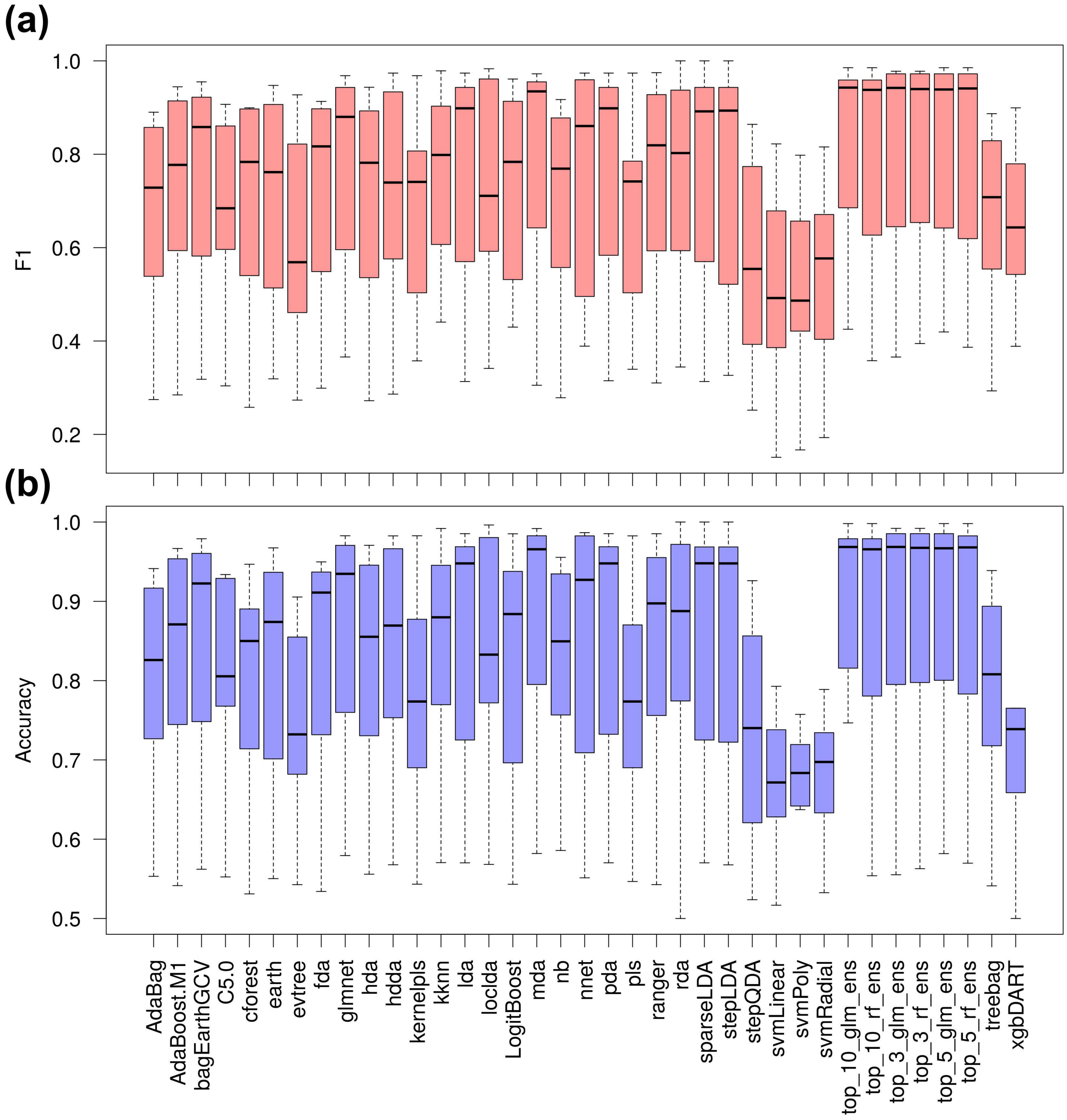

Figure S5. Distribution of (a) F1 and (b) balanced accuracy scores for every method among multi-class datasets. Methods are ordered alphabetically.

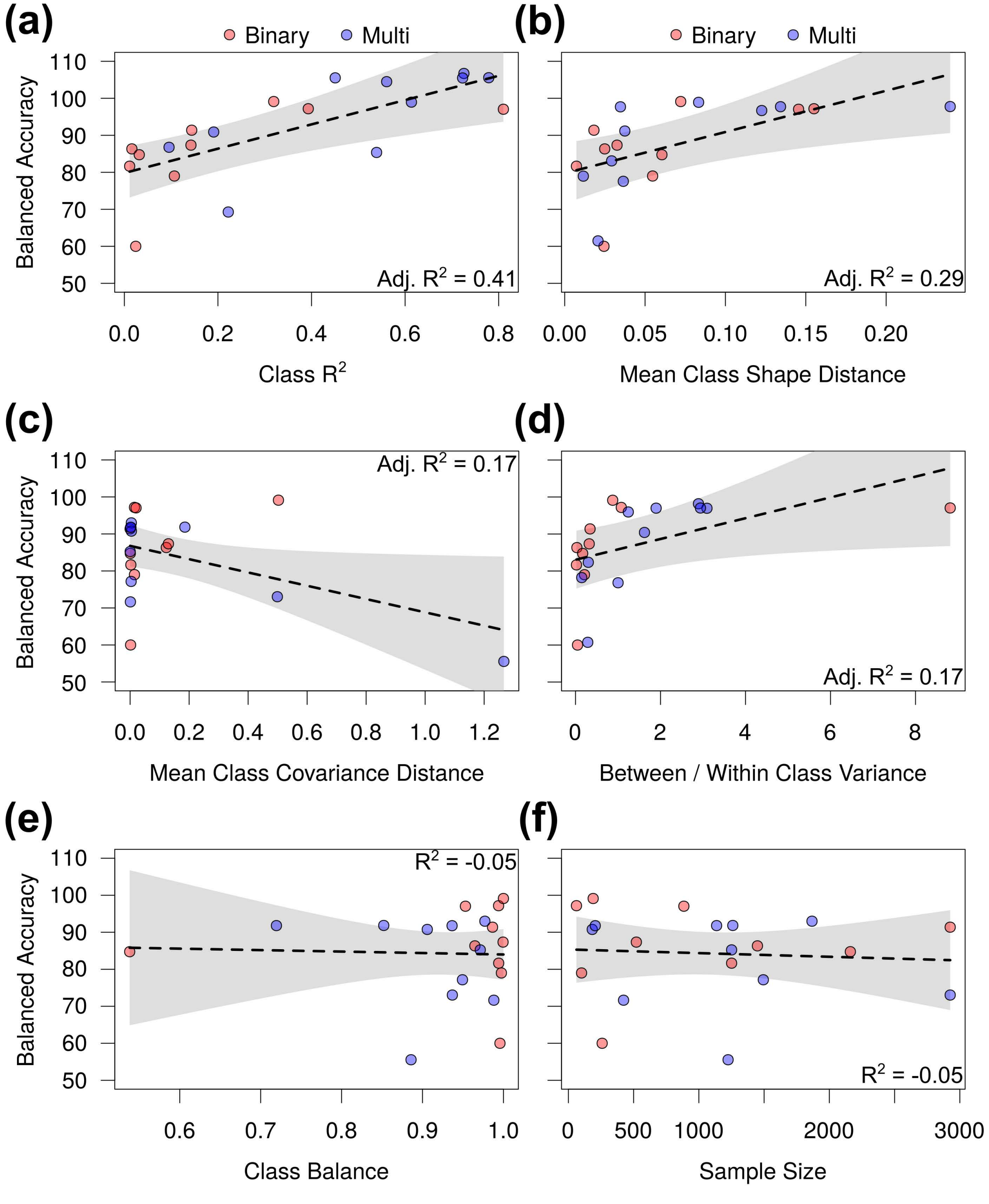

Figure S6. Balanced accuracy multiple regression plots that included a classification task covariate (red, binary; blue, multi) plus (a) class R^2, (b) mean class shape distance, (c) mean class covariance distance, (d) between- vs. within-class variance, (e) class balance, or (f) sample size explanatory variables. Lines of best fit with 95% confidence intervals are shown alongside model R^2 values.
